# Side-specific implicit training of attentional disengagement and reorienting

**DOI:** 10.1007/s00221-025-07049-0

**Published:** 2025-05-28

**Authors:** Karin Ludwig, Raffaela M. M. Böswald, Johannes Schusterbauer, Thomas Schenk

**Affiliations:** Clinical Neuropsychology, Department of Psychology, Ludwig-Maximilians-Universität München, Munich, Germany

**Keywords:** Attention, Disengage deficit, Disengagement deficit, Spatial neglect, Implicit learning, Spatial orienting paradigm

## Abstract

Some patients with parietal brain damage have problems disengaging their attention from ipsilesional stimuli. Although this disengage deficit has been described in the framework of the neglect syndrome, where it is particularly pronounced, no current treatment addresses this issue directly and side-specifically. In this study we propose a paradigm that could serve as such a training and – as a proof of concept – test its effects on the attentional allocation of healthy participants. We trained 36 participants between 20 and 35 years of age in a spatial orienting paradigm in which cues on one side were predictive of the opposite side, while the cues on the opposite side were neutral. We then compared reaction times before, during, and after this training. We could show that (1) this side-specific training specifically accelerated attentional disengagement from the predictive cues, (2) this effect persisted even when cues became unpredictive again, (3) it could be achieved implicitly, i.e., without the participants’ knowledge of the true cue-target-relationship, and that (4) it indeed relied on the learning of the cue’s predictiveness of the target location and not pure target occurrence probabilities. These results not only contribute to our knowledge about the spatial orienting of attention but might also form the basis for a new approach to treating the disengage deficit.

**Supplementary Information:**

The online version contains supplementary material available at 10.1007/s00221-025-07049-0.

## Introduction

**Fig. 1:**
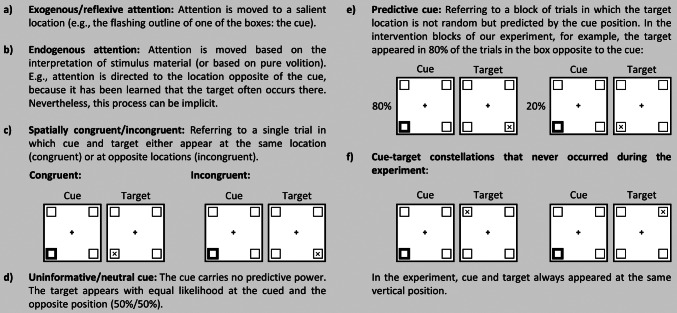
Basic terminology and design parameters of (our version of) the spatial orienting paradigm. (**a**) and (**b**) refer to the two types of attentional processes that can be assessed with this paradigm. (**c**) refers to the two different types of cue-target relationships that can occur in our experiment. In the literature, they are also called valid/invalid trials. For a more detailed explanation of why we refrain from using those terms, see our footnote about nomenclature (in the section ‘Present Study’). (**d**) and (**e**) differentiate cues based on their predictive value within an experimental block. (**f**) shows constellations that did not occur during the present experiment)

Orienting our attention according to external or internal demands is crucial to succeeding in activities of daily living. A particular deficit in this attentional orientation can occur after parietal damage, namely the disengage (or disengagement) deficit (Losier and Klein 2001; Posner et al. 1982, 1984; Ptak and Bourgeois 2024). It means that patients have problems disengaging attention from an ipsilesional location. Most often, it is described in the framework of the spatial orienting paradigm (Posner 1980). In this paradigm, salient spatial cues draw attention to their location. Targets that appear at this cued location are then processed faster than targets that appear at an opposite location. When patients with parietal lesions are performing this spatial orienting task, they show similar reaction time (RT) benefits after correctly cued targets (cue and target appear at the same position, see Fig. 1) as healthy participants, even if cue and target are presented in the contralesional hemispace. However, the patients’ performance worsens drastically when the cue has directed their attention to the (preferred) ipsilesional side, but the target appears at the contralesional side (Losier and Klein 2001; Posner et al. 1982, 1984; Ptak and Bourgeois 2024). This major performance deficit only appears one-sidedly, i.e., when disengaging attention from the ipsilesional side and moving it to the other side but not vice versa.

Morrow and Ratcliff (1988) found a relationship between the magnitude of the disengage deficit and neglect strength (conceptually replicated in Bonato et al. 2009) and that the reduction of neglect symptoms over time goes along with a decrease in the disengage deficit. This confirmed the preliminary results of Posner et al. (1982). In their review on the disengage deficit, Losier and Klein (2001) come to a similar conclusion: In groups of patients with a neglect diagnosis, the disengage deficit tends to be larger than in groups without. In their review on the topic, Ptak and Bourgeois (2024) even view the disengage deficit as “a functional marker of spatial neglect” (p. 1).

Interestingly, even though this disengage deficit was discovered decades ago (Posner et al. 1982, 1984) and seems to be at least a very common problem in patients with neglect (Bonato et al. 2009; Morrow and Ratcliff 1988; Posner et al. 1982; Ptak and Bourgeois 2024), none of the commonly used neglect treatments such as visual scanning therapy or prism adaptation (Kerkhoff and Schenk 2012) is theoretically based on this phenomenon.

In the current study, we present an experimental paradigm that aims to introduce a therapeutic intervention for the disengage deficit but also sheds light on attentional learning mechanisms in healthy participants. To our knowledge, our suggested training would be the only one specifically targeting covert attention (i.e., attentional shifts without eye movements) in neglect in a side-specific manner, i.e., covert attentional shifts toward the underattended side are trained while shifts towards the already overattended side are not.

### Training attentional disengagement and reorienting

In our proposed training, we aim to modulate the patients’ attentional disengagement in a spatial orienting paradigm (Posner 1980). When performing such a spatial orienting paradigm using a peripheral cue, two distinct attentional mechanisms can be observed depending on the predictiveness of the presented cues. If the peripheral cue is uninformative, our attention is only driven by the stimulus and is hence reflexively or exogenously oriented towards the position of the cue. In the case of a predictive cue, the first reflexive orientation towards the cue location will also happen but may be followed by a re-orientation towards a second position if that second position is indicated by the cue as the most likely location for an upcoming target (Chica et al. 2014; Egeth and Yantis 1997; Jonides 1981). Depending on the nature of the predictiveness, the targets might appear with above-chance probability at the location of the cue or the location opposite of the cue. For a more detailed description of cue types and attentional processes in the Posner paradigm in general and our experiment in particular, see Fig. 1.

Interestingly, neglect patients often show rather intact endogenous attentional reorienting when reacting to predictive cues even though their exogenous processes seem to be impaired severely (Bartolomeo et al. 2001; Làdavas et al. 1994; Luo et al. 1998). For example, Bartolomeo et al. (2001) compared healthy participants and neglect patients in a Posner task (Posner 1980) in which participants had been informed that 80% of the peripheral cues were predictive of targets on the opposite side. Hence, the best strategy for participants to succeed - once the cue had captured their attention exogenously - was to disengage attention from the cue and to endogenously orient their attention to the opposite side, making use of the predictive value of the cue. Indeed, the patients were able to do just that. These findings let us assume that training the process of endogenously disengaging and reorienting attention to the contralesional side could be used as an intervention to counteract some of the attentional deficits in neglect, especially the disengage deficit (Bartolomeo et al. 2001, 2007; Wansard et al. 2015).

### Side-specific predictiveness

The main idea of our new treatment approach is to use a spatial orienting paradigm (Posner 1980) and train patients to disengage attention quickly from ipsilesional cues (since they predict the contralesional side) and move it to the contralesional (usually neglected) side. In contrast to previous studies (Bartolomeo et al. 2001, 2007; Siéroff et al. 2007; Wansard et al. 2015), this intervention should function in a side-specific manner, as disengagement from the contralesional side is not impaired (Posner et al. 1984) and thus does not need to be trained.

In the present study, we want to test – in healthy participants – whether endogenous cueing effects based on the predictiveness of the cue can be established even when only cues on one side are predictive. We want to explore whether this side-specific predictiveness can be learned in an intensive training and how long the effect persists when cues again become unpredictive. If the training and the subsequent transfer of this side-specific predictive cueing effect are successful, the training might function as a neglect intervention to alleviate the disengage deficit. The side-specificity is an important aspect of the intervention because, firstly, neglect is characterized through a spatial imbalance of attention in favour of the right hemispace (Hilgetag et al. 2001; Kinsbourne 1987). Hence, training the patients side-specifically might help to reduce this imbalance and its negative consequences. Secondly, methodologically speaking, a side-specific training predicts an asymmetry and thus provides a strong test for the effect of the training. Namely, it makes a qualitative, specific prediction and allows for within-comparisons at the same time point, thereby avoiding potential confounding factors such as unspecific practice effects. Therefore, side-specificity also serves as a quality marker of our experimental design.

### Duration of the learning effects

Regarding long-term learning effects of predictive cueing paradigms, it remains unclear how long such effects of learned predictiveness persist in uninformative settings, i.e., settings in which the cues no longer predict the target location (see Fig. 1d). So far, previous studies indicate that a transfer (of learned predictiveness) might be possible in healthy participants (Hughes 1984; Rieth and Huber 2013). However, they do not answer the question of the duration of such transfer effects. To assess the training’s applicability as an intervention and its promise as a therapy with long-lasting benefits, it is important to do just this. A training effect that lasted only for a short time and disappeared rapidly after exposure to neutral trials would be of little therapeutic value. Therefore, assuming that a side-specific predictive cueing effect can be produced in our training phase, we want to test how long this potential transfer effect persists in an uninformative setting. To do this, we will split our posttest into two subblocks and examine whether the effect can be observed in both subblocks or whether it degrades with time.

### Implicit training

In our study, we will not inform our participants about cue predictiveness, i.e., in how many trials cue and target appear at the same location and in how many trials their locations differ. We thus aim at implicit learning of the cue-target contingencies.

Neglect patients often lack insight into their disorder – a phenomenon known as anosognosia (Grattan et al. 2018), which can result in reduced therapeutic compliance and training motivation. Hence, interventions that are based on the idea of training patients to *voluntarily* adopt new (attentional) strategies might fail if the participants do not cooperate and thus do not engage in the planned strategy. The effects of an implicit training will hopefully be less affected by anosognosia and show better transfer from the training to everyday life.

Various studies indicate that healthy participants show predictive cueing effects independently of whether they have previously been informed about the cue predictiveness or not (Bartolomeo et al. 2007; López-Ramón et al. 2011; Risko and Stolz 2010) and even if they could not explicitly report the cue-target contingencies (Bartolomeo et al. 2007). The latter has also been shown in neglect patients (Wansard et al. 2015).

### Present study

We trained our participants with cues that predicted target occurrence at the opposite side in a discrimination task and then compared the speed of their attentional disengagement and endogenous reorienting of covert attention before (pretest) and after (posttest) the training. To be exact, the training phase (intervention) consisted of predictive cues on one side and uninformative cues on the other side. Thus, on one side of the display, participants were trained to disengage and endogenously reorient their attention. In contrast, on the other side, attentional orientation was driven purely by the exogenous cues.

With our specifically adapted paradigm (see Sect. “Stimuli and task”), we hoped to answer the following three questions: Firstly, we wanted to explore whether cue predictiveness could be learned side-specifically and thus lead to an improvement of attentional disengagement from one and reorienting to the other side of space.

Secondly, we asked ourselves whether the learned one-sided predictive cueing effect of the intervention block could be transferred into a non-predictive setting or whether the training benefit diminished once the cues lost their predictive meaning.

Thirdly, we wanted to explore to what extent the attentionally more challenging side-specific predictive cueing effect was learned implicitly (our participants were not informed about cue predictiveness since we wanted to foster implicit learning processes) or whether explicit components of perception might also be responsible for some of the observed effects (see Supplement).

Lastly, in our control analyses (see Supplement), we tested whether the results indeed relied on learning cue predictiveness and if we could thus exclude an alternative explanation.

## Materials and methods

### Participants

To find a minimum effect of *d* = 0.50, which was found to be the smallest effect size of interest in health-related quality of life changes (Norman et al. 2003) with a power of 80% and a significance level of α = 0.05, a minimal sample size requirement of *N* = 34 was calculated using G-power (Version 3.1.9.2; Faul et al. 2007). Based on the recommendations of Chica and colleagues (2014), in the end 36 participants (24 female, 24 right-eye dominant) at the age of 20–35 years (*M* = 22.92, *SD* = 3.24) were tested in our study. All participants were right-handed, naïve to the purpose of the study, and had normal or corrected-to-normal vision. All participants gave their written informed consent to participate in the study and for their anonymized data to be shared. Participants either received monetary compensation of 9 € per hour or course credits. The study was approved by the local ethics committee of the psychology department of the Ludwig-Maximilians-Universität München.

### Apparatus

Participants were tested individually while sitting in front of a BenQ XXL2420Z, 24” monitor with a screen resolution of 1920 × 1080 pixels and a refresh rate of 144 Hz. A viewing distance of 92.5 cm was fixed via a chin and head rest. During the experiment, we tracked the participants’ dominant eye (measured via the whole-in-the-card test beforehand) monocularly, using an Eyelink 1000 Plus (SR Research, Mississauga, Ontario, Canada) with a temporal resolution of 2000 Hz. Participants’ responses were recorded on a PST Serial Response Box. The stimuli were created using MATLAB R2015a (MathWorks, Natick, MA) and the Psychophysics Toolbox Version 3 (Brainard 1997; Kleiner et al. 2007; Pelli 1997).

### Stimuli and task

We used the experimental paradigm introduced by Bartolomeo et al. (2001), i.e., a spatial orienting paradigm with predictive cues, but added several changes to make it more suitable for our research question and our current sample of healthy participants.

In neglect patients, the side with the cues that predict the opposite side would of course be the ipsilesional (usually right) side to train attentional orientation towards the neglected (usually left) side. In the current study with healthy participants, however, it was the other way around to take advantage of pseudoneglect, an attentional left side advantage typically found in healthy participants (Bowers and Heilman 1980; Jewell and McCourt 2000; Ludwig and Schenk 2022; McCourt 2001). Thus, the predictive cues in the intervention block of this study appeared on the left side (and predicted the right side), aiming to speed up disengagement from the left side and facilitate endogenous orienting to the right side of the screen.

Furthermore, our participants performed a discrimination instead of a detection task as we wanted to delay a potential onset of inhibition of return (IOR), which starts later in discrimination than in detection tasks (Chica et al. 2006, 2014). This was done to provide enough time for the endogenous reorienting of attention that we wanted to manipulate with our predictive cues but which is a slower process than exogenous orienting and, thus, requires a longer SOA to be applied successfully (Chica et al. 2013; Müller and Rabbitt 1989).

On top of that, stimuli could not only appear at two (left or right) but at four (top-left, top-right, bottom-left, bottom-right) positions. The target that followed a cue on the top-left, for example, always appeared either at the top-left or at the top-right (see Fig. 1). The aim of this alteration was to increase the chances that participants would react according to cue predictiveness and not according to target side regularities.

All stimuli in the discrimination task were presented in light grey (RGB: all 128) on a black background. Participants responded to the target – an ‘X’ (size: 0.50° x 0.52° visual angle) or an ‘O’ (size: 0.50° x 0.52°) – by either pressing the left or the right button of the response box while maintaining gaze fixation on a 0.4° x 0.4° fixation cross in the center of the screen. The button-‘target type’-correspondence (e.g., left for ‘X’, right for ‘O’) was randomly counterbalanced between participants and kept constant throughout the whole experiment. ‘X’ and ‘O’ appeared equally often in the different conditions so that possible effects of ‘target side/response side correspondence’ would average out without systematically influencing the results.

**Fig. 2:**
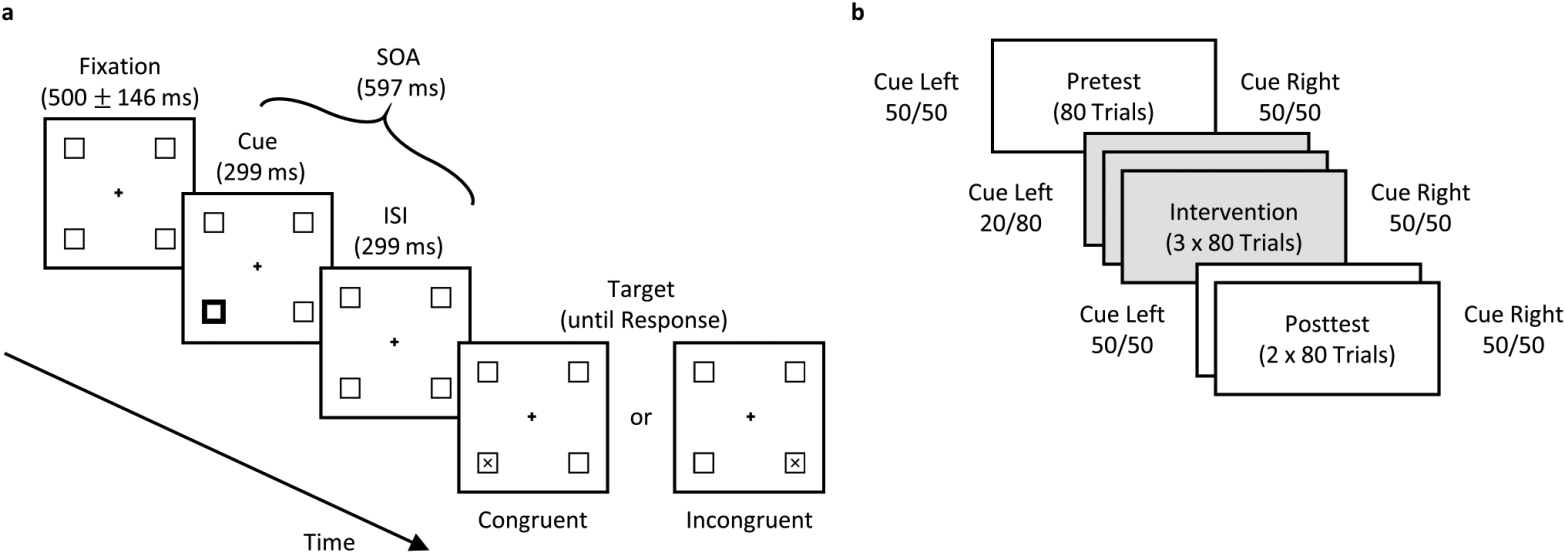
Exemplary trial of the posner discrimination task (**a**) and sequence of experimental trials (**b**). (**a**) In the experiment, stimuli were light grey on a black background and otherwise as depicted here. After a fixation period, a cue (a short highlighting of one of the four boxes) appeared. Subsequently, a blank interval (identical to the fixation screen) was shown. Afterwards, a target– either an “X” or an “O”– was displayed either in the cued box (spatially congruent trial) or the horizontally opposite box of the cued box (spatially incongruent trial). Cue and target locations did not differ vertically. Participants had to indicate the perceived target (X (depicted) vs. O (not depicted)) by pressing the corresponding button. (**b**) Participants carried out a pretest with uninformative cues (i.e., 50/50: targets appeared in 50% of the trials on the side of the cue (spatially congruent) and in 50% on the other side (incongruent), followed by the intervention phase with left-sided predictive cues (20% spatially congruent, 80% incongruent) and right-sided unpredictive cues. Afterwards, a posttest with uninformative cues followed. Participants did not know about different experimental blocks, nor a change of cue predictiveness within the blocks

Each trial started with a 500 ms (± up to 146 ms) fixation period (see Fig. 2). The screen of the fixation period only contained the central fixation cross and four surrounding squared boxes (size: 1.15° x 1.15°). The distance from the fixation cross to the center of each squared box was 2.708°. During this period, the accuracy of the participant’s central fixation on the cross was checked. If, after 2 s, the participant’s gaze position was still not within a 1 × 1° fixation window, the trial was interrupted, and a recalibration was conducted. If fixation was accepted, a cue appeared for 299 ms. This cue was displayed as a thickening of the outline width of one of the boxes from a pen width of 0.03° to 0.20°. Hence, it was perceived as a quick flash or highlighting of one box. After the cue offset, there was a screen identical to the fixation screen for another 299 ms (interstimulus Interval, ISI), followed by the appearance of the target letter (SOA = 597 ms). The target could appear either in the previously cued box (spatially congruent trial) or the horizontally opposite box of the cue (spatially incongruent trial^1^).

There were no trials in which the cue and target position differed vertically (see Fig. 1). The target remained on the screen until participants identified the target type via button press. Participants were instructed to fixate the fixation cross in the center of the screen (hence, the task was a covert attention task).

### Procedure

Before the experiment, a nine-point calibration-validation process was conducted. This was repeated during breaks within the experiment, if necessary, and whenever calibration was no longer satisfactory during experimental trials. Calibration was considered satisfactory if the deviation of calibration and validation gaze positions was below 1° visual angle for each of the nine locations and if the average deviation of all positions was below 0.5°. As soon as calibration was accepted, participants performed 16 practice trials followed by 480 experimental trials (see Fig. 2b). The experimental trials were separated into three blocks: a pretest (80 trials), an intervention block (240 trials), and a posttest (160 trials). The posttest was twice as long as the pretest to be able to observe potential changes in our effect over time. The first five participants conducted 280 trials in the intervention block, resulting in a total of 520 experimental trials to be performed. We chose to shorten the experiment for the remaining participants to prevent fatigue, which can occur in cases of long attentional tasks (Chica et al. 2014). In the pre- and posttest, the cues were equally likely to be spatially congruent or incongruent and hence the cue contained no information concerning the target side. However, during the intervention, side-specific predictiveness was trained, which is why left-sided cues predicted a target on the right side in 80% of the trials (80% spatially incongruent cues) while right-sided cues remained uninformative (50/50). For the first five participants, the 80/20 ratio applied to the whole intervention block but not to each individual intervention subblock (Int1, Int2, and Int3) since the split of the intervention unit into three subblocks was carried out after the trial sequences had been generated. For the remaining 31 participants, cue predictiveness in all subblocks was as indicated.

Participants were not informed about the existence of different cue-target relationships or changes in experimental blocks. Every 34 trials, a break was automatically initiated. Participants could rest for as long as they wanted. There were no additional breaks marking the end/beginning of experimental blocks: each of the experimental blocks directly followed the previous. After the experiment, participants filled out a debriefing questionnaire (see Supplemental Table 1) to report their perception of cue predictiveness and target side occurrence during the experiment. The participants were first asked what they assumed to be the purpose of our experiment. Further, they had to state whether they had noticed anything specific concerning the statistical relationship between the cue- and target side. Next, they were asked whether the cue had predicted the same or the opposite target side more often; after that, they had to estimate the proportion of spatially incongruent cues. Those questions were later used to assess the implicitness of our intervention. Lastly, participants were asked to estimate the proportion of right-sided and left-sided targets throughout the whole experiment. The complete questionnaire in German (original) and English (translation) can be found in Supplemental Table 1.

### Data analysis

For the behavioral data analysis, mean RTs as well as double-difference values (DD) of mean RTs were evaluated. The DD value was calculated as follows:

$$\eqalign{ DD\, & = \left( {R{T_{Cue\>left,\>\>incongruent\>}} - \>R{T_{Cue\>left,\>\>congruent}}} \right) \cr & - (R{T_{Cue\>right,\>\>incongruent\>}} - \>R{T_{Cue\>right,\>\>congruent}}) \cr} $$

Both single differences each compare RTs for spatially incongruent and spatially congruent trials at one specific cue side (lateral cueing effect), while the DD value compares the magnitude of these lateral cueing effects for left-sided versus right-sided cues. Hence, regarding attentional orienting mechanisms, the DD-value quantifies the asymmetry in endogenous attentional orienting between left-sided versus right-sided spatially incongruent cues.

Thus, a negative DD value in our experiment would represent a faster endogenous attentional orienting after left-sided than after right-sided spatially incongruent cues. In line with our hypothesis, we predicted that the DD value should decrease from pretest to intervention (or from pre- to posttest, respectively).

RTs were the dependent variable in a prerequisite check (independent variable: spatially congruent vs. incongruent) and in the main hypothesis check (can side-specific cue predictiveness be learned?), which was carried out with a 4 × 2 × 2 three-way repeated measures ANOVA with the factors ‘Time’ (Pre, Int1, Int2, Int3), ‘Cue Side’ (left, right), and ‘Spatial Congruency’ (incongruent, congruent), expecting a significant three-way interaction. The ANOVA was followed up by two separate ANOVAS for left-sided and right-sided cues and by comparing DD values (dependent variable) in the pretest with the DD values of each intervention block (Int1, Int2, Int3). In case of sphericity violations, Greenhouse-Geisser corrected degrees of freedom and p-values are reported.

To determine whether the effect carried over to a non-predictive setting, we compared DD values (dependent variable) in the pretest with those in the posttest (and separately for both subblocks of the posttest to see how long the effect survived) with repeated measures t-tests (and additional Wilcoxon signed-rank tests in case of violation of the normality assumption).

To test whether our manipulation had been noticed or whether the effects were based on implicit processes, we tested whether participants could correctly guess after the experiment whether the cue more often predicted the same (1) or the opposite side (2) for target appearance or whether it was balanced. To this aim, we conducted one sample Chi-square test on the frequencies for (1) vs. (2). We further compared the participants’ estimates (in how many trials (%) did the target appear opposite to the cue? ) to 50% via a Wilcoxon signed-rank test (the normality assumption had been violated) and a Bayesian one-sample Wilcoxon signed-rank test. These and a further analysis including only participants who had incorrectly guessed the proportion of spatially incongruent trials can be found in the Supplemental Analysis 1.

Lastly, we performed a 2 × 2 ANOVA on mean RTs (dependent variable) with the independent factors ‘Time’ (Pre, Int) and ‘Target Side’ (left, right) based only on trials with right-sided cues to rule out the potential that participants had just learned that a target was (slightly) more likely to appear on the right side of the screen and not cue predictiveness (see Supplemental Analysis 2).

Whenever experimental blocks were divided into subblocks, and more than one paired comparison was conducted, Bonferroni correction was used. For easier understanding, the reported p-values are already Bonferroni corrected (e.g., multiplied by the number of comparisons), if necessary.

The data were analyzed using R, version 4.2.1 (R Core Team 2022). All anonymized data, analysis code, and research materials are available at https://osf.io/brzpg/.

## Results

Only trials with correct responses and good fixation (classified by visual inspection of the Eyelink data) were included in the analyses. Of the remaining data, RTs below 150 ms (anticipatory response) or above three standard deviations of the mean RT of every participant grouped by spatially congruent and incongruent trials were excluded. In total, 5.65% of all trials were removed due to incorrect responses (1.27%), detected eye movements (3.12%), or outliers (1.26%).

### Prerequisite

**Fig. 3:**
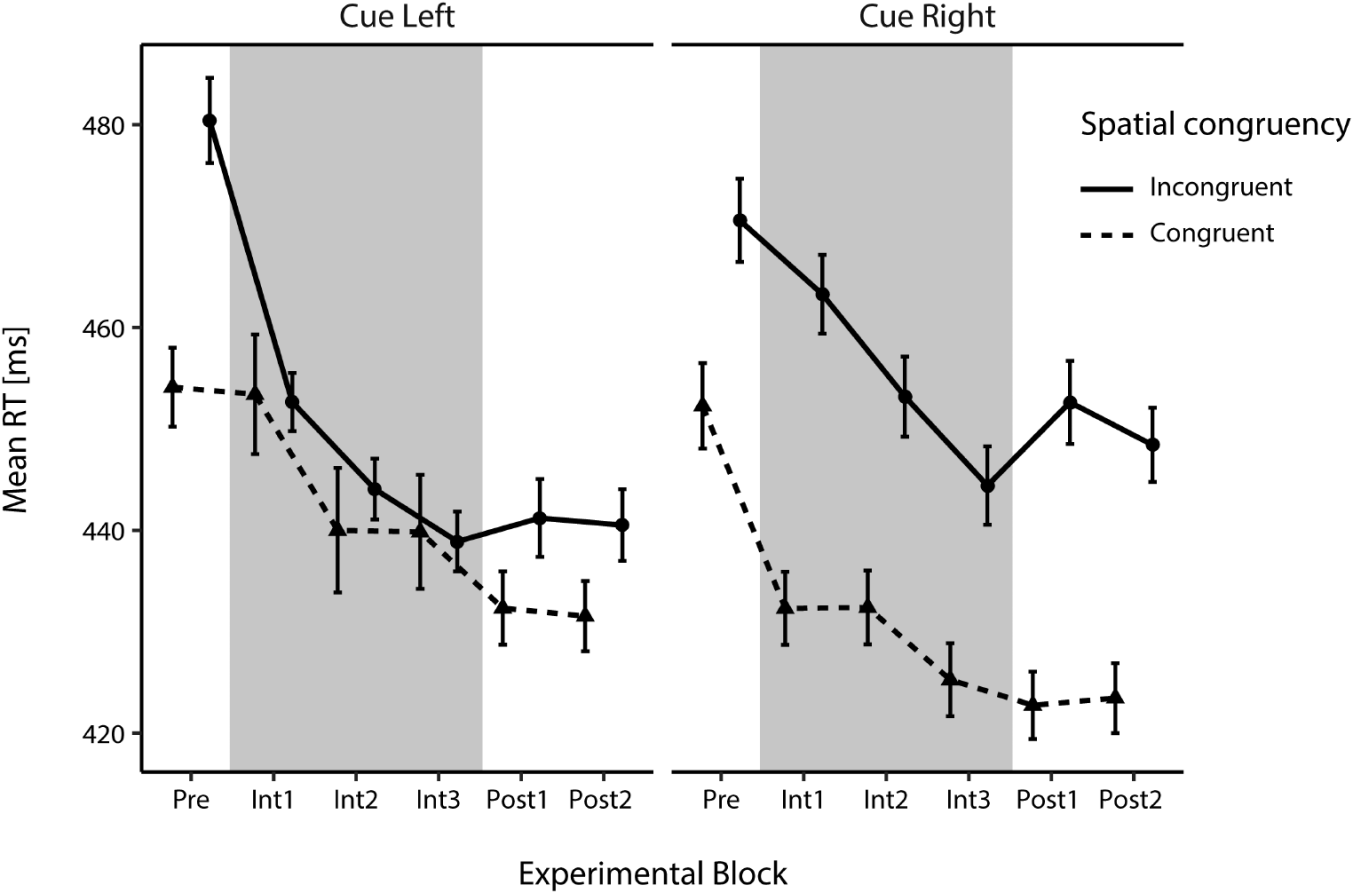
Mean reaction times (RTs) over the course of the experiment, i.e., in the pretest (Pre), the three intervention subblocks (Int), and the two posttest subblocks (Post) split by the factors cue side and spatial congruency. Note that the y-axis starts at 420 ms. Error bars depict the standard error of the mean

For the pretest data, we could confirm the existence of a highly significant Posner effect (*t*(35) = 5.51, *p* <.001, *d* = 0.92), resulting in faster RTs for spatially congruent (*M* = 453.19 ms, *SD* = 105.24) than for incongruent trials (*M* = 475.56 ms, *SD* = 106.54), see Fig. 3. This shows that the cue draws attention exogenously, which means that its predictiveness can potentially be learned, which is a prerequisite for the further analyses.

### Learning of cue predictiveness

First, we wanted to explore whether participants could learn the one-sided predictiveness of cues in the intervention block and react accordingly.

Our three-way repeated-measures ANOVA with the factors ‘Time’ (Pre, Int1, Int2, Int3), ‘Cue Side’ (right, left), and ‘Spatial Congruency’ (congruent, incongruent) revealed, as expected, a main effect for ‘Time’ (*F*(2.2,77.0) = 9.36, *p* <.001, η_p_^2^ = 0.21) as well as for ‘Spatial Congruency’ (*F*(1,35) = 25.78, *p* <.001, η_p_^2^ = 0.42), which is in line with an improvement over time and faster RTs after spatially congruent trials in general. The ANOVA also showed a significant two-way interaction effect of ‘Cue Side’ and ‘Spatial Congruency’ (*F*(1,35) = 10.40, *p* =.003, η_p_^2^ = 0.23) and – crucially – a significant three-way interaction of the factors ‘Cue Side’, ‘Time’ and ‘Spatial Congruency’ (*F*(3,105) = 5.96, *p* <.001, η_p_^2^ = 0.15). To follow up on the significant three-way interaction, we carried out two two-way repeated ANOVAs with the factors ‘Time’ (Pre, Int1, Int2, Int3) and ‘Spatial Congruency’ (congruent, incongruent) – one for trials with cues on the left, one for trials with cues on the right. As expected, the interaction was significant for the ANOVA with the cue-left-trials (*F*(2.3,80.7) = 5.44, *p* =.004, η_p_^2^ = 0.13) but not for cue-right-trials (*F*(2.3,79.8) = 1.44, *p* =.24).

These interactions support our hypothesis that the cue’s spatial congruency affects RTs differently after left-sided than right-sided cues and that this effect significantly changes over time. In other words, we indeed found the expected modulation by left-sided predictive cues, indicating that the participants reacted to the new cue-target contingency (i.e., cue predictiveness). This pattern can also be seen in Fig. 3, displaying that RTs to targets that appeared after the unpredictive cues on the right decreased over time, consistent with a general effect of improvement over time. On top of that, a pronounced spatial congruency effect can be detected, i.e., when cue and target appeared at the same location (congruent), RTs were faster than when they appeared at opposite locations (incongruent). This effect was very stable over all blocks. Interestingly, compared to this stable congruency effect over time, in trials with cues on the left (left panel in Fig. 3), a strong modulation of RTs by our experimental manipulation of cue predictiveness can be seen: RTs to targets on the right (i.e., spatially incongruent to the cue but expected at this location, since this combination was present in 80% of trials; left panel, continuous line in Fig. 3) became faster, which is in line with our ANOVA results and our prediction that participants can use the cue’s predictiveness to faster disengage their attention from the cue on the left and move it to the target on the right. At the same time, RTs to targets on the left (i.e., spatially congruent, 20% of trials, left panel, dashed line in Fig. 3) improved much less over the course of the intervention, indicating that the predictiveness of the cue was picked up by the attentional system of our observers and influenced their behavior.

**Fig. 4:**
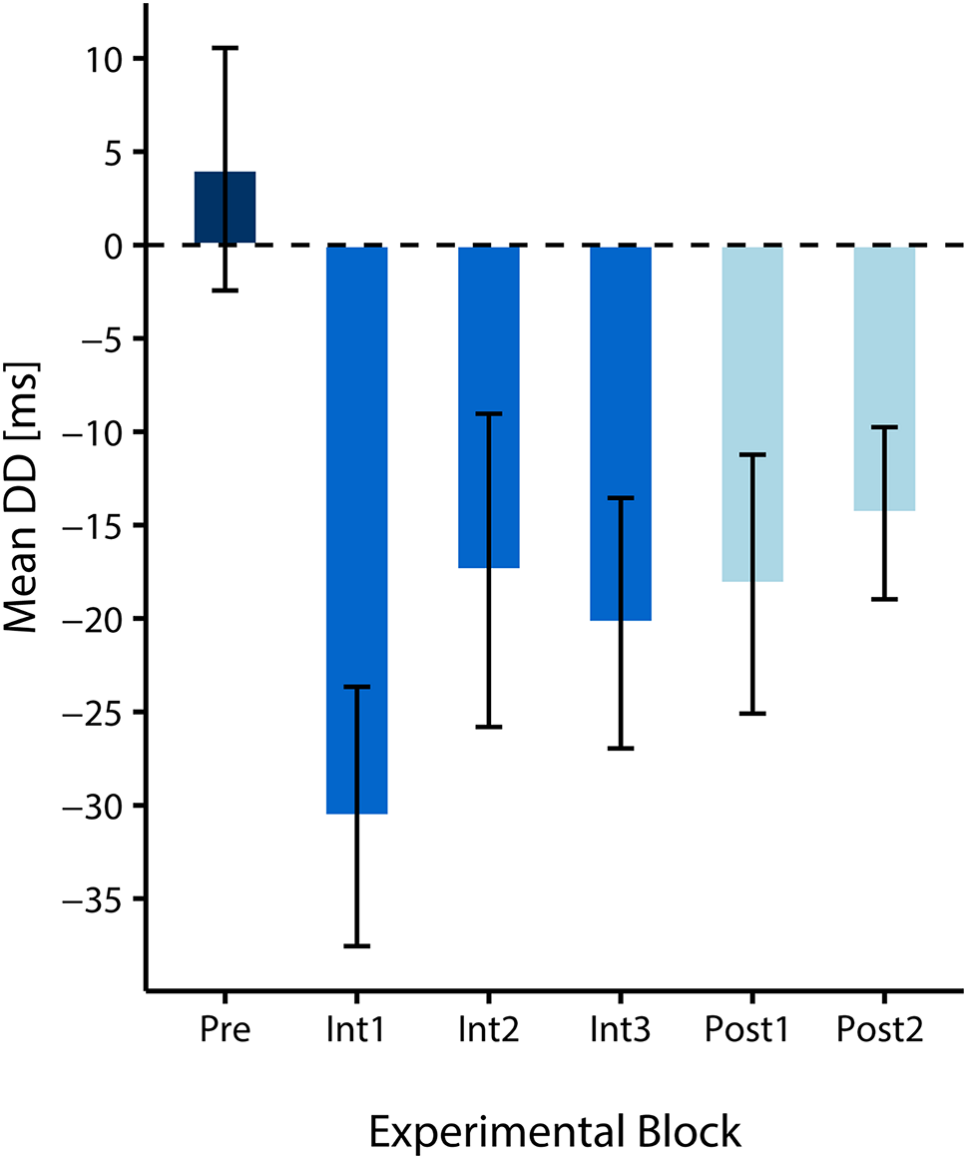
Mean double-difference values (DD) in milliseconds during the experimental phases (Pre, Int1, Int2, Int3, Post1, Post2). Error bars depict the standard error of the mean

To assess this further, we examined how long it took for the participants to internalize the statistical predictiveness of left-sided cues and use it to speed up their RTs following left-sided spatially incongruent cues. Hence, we compared DD values in the pretest with DD values of each subblock of the intervention phase. Here, we expected the effect to increase with each block as information about the cue-target correspondence accumulates over time. For a depiction of the DD values in each experimental block, see Fig. 4. The data based on which the DD values were calculated are plotted in Fig. 3.

All Bonferroni corrected comparisons of the DD value in the pretest (*DD*_Pre_ = 4.05, *SD*_Pre_ = 38.97) with the DD values in each subblock of the intervention (*DD*_Int1_ = -30.60, *SD*_Int1_ = 41.65; *DD*_Int2_ = -17.42, *SD*_Int2_ = 50.31; *DD*_Int3_ = -20.25, *SD*_Int3_ = 40.25) reached significance (Int1: *t*(35) = 4.82, *p* <.001, *d* = 0.80; Int2: *t*(35) = 2.99, *p* =.008, *d* = 0.50; Int3: *t*(35) = 2.6, *p* =.020, *d* = 0.43). Surprisingly, the strongest left-sided cueing effect was found in Int1. Accordingly, mean RTs after spatially incongruent left-sided cues were already faster than mean RTs after spatially congruent left-sided cues in Int1 (see Figs. 3 and 4), which is surprising considering that by this time, participants had not experienced many trials with the predictive left-sided cues. Namely, in Int1 (as in Int2 and Int3), each participant was presented with 32 spatially incongruent left-sided cues (out of 40 left-sided cues). We tested whether this constellation was somehow created arbitrarily by our outlier exclusion criteria (i.e., whether incongruent trials with left-sided cues in Int1 were particularly prone to be removed thus shortening the average RT), which was not the case. This early appearance of the effect can be either considered as a fluke finding or as evidence that the cue predictiveness was detected and learned quickly.

Interestingly, although the predictiveness of the cue had the expected outcome, the prediction effect did not completely override the exogenous cueing effect but was rather additive: RTs to spatially incongruent but correctly predicted targets (80%) did not become faster than RTs to spatially congruent but incorrectly predicted targets (20%). However, this might happen after longer practice.

### Transfer of learned cue predictiveness

Having confirmed that a side-specific improvement of endogenous attentional reorienting was possible in a setting in which predictive cues were present, we wanted to examine whether this side-specific predictive cueing effect transferred to a non-predictive setting and, in case of transfer, how stable the effect would be.

The comparison of the RT data in the pre- and the posttest revealed that DD values were still significantly reduced in the posttest (*DD*_Post_ = -16.68, *SD*_*Post*_ = 29.17) compared to the pretest (*DD*_Pre_ = 4.05, *SD*_*Pre*_ = 38.97; *W* = 497, *p* =.005, *r* =.44; *t*(35) = 2.82, *p* =.004, *d* = 0.47), also see Fig. 4. Again, Negative DD values (as in the posttest) indicate that attentional disengagement and reorienting after spatially incongruent left-sided cues is faster than after spatially incongruent right-sided cues.

Next, when assessing the development of the transfer effect, the comparison of DD values Pre (see above) vs. Post1 (*DD*_Post1_ = -18.16, *SD*_Post1_ = 41.59) and the comparison Pre vs. Post2 (*DD*_Post2_ = -14.36, *SD*_Post2_ = 27.64) reached significance (Post1: *W* = 476, *p* =.024, *r*_*B*_ = 0.38; *t*(35) = 2.30, *p* =.028, *d* = 0.38; Post2: *t*(35) = 2.92, *p* =.006, *d* = 0.49), see Fig. 4. Thus, our data support the hypothesis that faster attentional disengagement and reorienting due to a side-specific training with one-sided predictive cues can be transferred to and maintained (for at least 160 trials) in a non-predictive setting. The development of DD values throughout the whole experiment (and of the RTs on which they are based) can be seen in Fig. 4 (and Fig. 3). They show that the side-specific predictive cueing effect is maximal during the intervention but only marginally smaller during the posttests.

### Implicitness of the effect

As a next step, we examined whether the one-sided predictiveness of the cues was learned implicitly or whether an explicit perception of the cue predictiveness was necessary to produce the effect. To this end, we tested on data from the debriefing questionnaire whether participants had noticed that cues had predicted the spatially incongruent position more often than the congruent one (which would have enabled an explicit strategy), see Supplemental Analysis 1. The results gave us no reason to assume this and a further analysis including only participants that had guessed the opposite or assumed that cues were uninformative (also see Supplemental Analysis 1) showed the same result as the main analysis: side-specific cue predictiveness was learned. We conclude that the detected predictive cueing effect was most likely based on implicit learning.

### Control analysis: cue predictiveness vs. target occurrence

Since the main aim of this study was to find out whether healthy participants can learn about one-sided cue predictiveness and use it to improve their attentional disengagement from one specific side, the interpretation of our results relies heavily on the fact that participants truly used the predictiveness of left-sided cues (of targets on the right) and not the slight majority of right-sided targets to guide their attention. In Supplemental Analysis 2, we could show based on trials with right-sided cues (which were always uninformative) that the former was indeed the case and that the effect did not rely on learned target occurrence statistics.

## Discussion

The present study provides the first evidence that participants’ endogenous attentional orienting can be modulated side-specifically by training them with one-sided predictive cues in a spatial orienting paradigm (Posner 1980). Participants were able to learn the cue predictiveness, which led to faster side-specific attentional disengagement from the predictive cues. Importantly, the effect also transferred to a subsequent non-informative posttest and survived until a second posttest, indicating that the effect might not be short-lived but could be maintained despite the change of cue-target contingencies. Further, our findings suggest that even a quite complex regularity can be extracted automatically from the sensory information and shape our attentional performance without observers becoming aware of the relationship. Lastly, our control analysis suggests that the effect was due to learned cue predictiveness and that participants did not just orient their attention towards the right side of the screen without reacting according to the cue (for more detailed information, see Supplemental Analysis 2).

First, we tested whether healthy participants could learn about one-sided cue predictiveness during an intensive training phase (intervention) and, consequently, improve their attentional disengagement from this specific side. Since participants showed a typical Posner effect (faster RTs in spatially congruent than in incongruent trials) in the pretest, deviations from this effect can be attributed to the predictiveness manipulation in the intervention phase. Our results indicate that our participants learned about the cue predictiveness during the intervention phase and were able to use it to speed up their attentional disengagement and their endogenous attentional reorientation to the predicted side of the screen. This is in line with previous data from healthy participants, as well as neglect patients, who were able to learn about and respond appropriately to predictive cues on both sides of the screen (Bartolomeo et al. 2001, 2007; López-Ramón et al. 2011; Wansard et al. 2015). In our study, we extend these findings by showing that it is possible to use those predictive cues also side-specifically and train attentional disengagement from only one side. This finding is of special interest, as it goes in line with methodological as well as potential therapeutic advantages. Training participants side-specifically lets us predict a lateral asymmetry in attentional processes and RT data. This asymmetry can then be tested within participants, reducing possible confounding factors like unspecific training effects and between-person differences. Furthermore, side-specific attentional training might provide the opportunity to counteract lateral spatial biases such as in the neglect syndrome. As mentioned earlier, due to their brain lesion, neglect patients have difficulties disengaging attention from stimuli in the right hemispace, which prevents them from orienting attention to the left hemispace. Thus, training their attentional disengagement side-specifically could redress this attentional imbalance and function as a therapeutic intervention to reduce some of their main deficits.

Regarding the applicability and usefulness of this intervention for therapeutic purposes, other studies indicate that such learning effects based on spatially incongruent cue-target relationships (irrespective of the mechanisms underlying those effects) might further intensify with prolonged training time or multiple training days. Warner et al. (1990) showed that after a multi-day training with cues that predicted the opposite side, participants even showed RT benefits at extremely short SOAs (< 150 ms). Further, Rieth and Huber (2013) observed that after two blocks of exposure to cues that predicted the opposite side, their participants showed predictive cueing effects at SOAs of 200 ms. Normally, only reflexive (exogenous) attentional orientation can operate within such short intervals (Chica et al. 2014; Posner et al. 1982). We used fairly long SOAs (597 ms) to achieve our side-specific effects. But presumably more intense training (e.g., more intervention trials or more intervention days) combined with adaptively decreasing SOAs might allow us to achieve even faster attentional reorienting. This would also be important for the application in neglect patients, since the faster the reorienting to the ipsilesional side, the larger the potential benefits in activities of daily living could be.

When assessing the applicability of our paradigm for a potential therapeutic intervention, another important factor to be examined is whether the training effects transfer to a neutral attentional task and whether such transfer effects can be maintained for more than a few trials. This question is of special interest as it is important for a training effect not to be limited to the training duration but also to influence behavior afterwards. Many current treatments for spatial neglect lack a proper transfer effect into daily life (Kerkhoff and Schenk 2012); a first step to improve transfer into the patients’ daily life is ensuring that the training effects outlast the training period itself. Fortunately, our results show that the training effects were still found in posttests that no longer provided a training stimulus. Even in the second posttest (Post2), which took place after 80 trials without any cue-target information, the lateral predictive cueing effect could still be detected. On top of that, looking at the effect size in the first and second posttest blocks, it seems like the effect did not diminish during Post2. Instead, the aftereffects of the spatial contingency learning seemed to be even stronger in Post2 than in Post1. This lets us believe that the predictive cueing effect indeed lasted for a minimum of 160 trials. Thus, the results of our study point to the fact that the training effect not only has the potential to transfer to neutral settings but also to remain stable for at least a short while. This is one of the prerequisites for a successful transfer of the effect beyond the training phase itself. In future studies, it would be interesting to address two further questions. Firstly, we would like to assess which training duration leads to which duration of the effects and if a sustained response can be achieved with an increased training duration. Secondly, we would like to examine whether the created training effect can also transfer to different contexts, e.g., a different task or a similar task with different stimuli to increase its potential for a future therapeutic intervention.

Besides the mere existence or significance of an initial transfer effect, another aspect that should be considered when assessing the potential impact of our side-specific training in daily life is the size of the effect. Even with a single training session of under an hour, we achieved a medium effect size. We believe that with more intervention trials during one session (Rieth and Huber 2013) or with various subsequent training days (Warner et al. 1990), the effect size could be even further increased. Quite possibly, an effect size estimate from a study with patients might also be higher since patients – as opposed to our healthy participants – have something to gain from this training.

We also examined whether side-specific predictiveness could be learned implicitly – without our participants being informed about it or being able to explicitly perceive and verbalize the regularities. In general, our results suggest that our participants were not aware of the cue predictiveness while still showing a clear attentional bias. Our control analyses (for more details, also see Supplemental Analysis 1) confirm previous findings that learning about and responding to predictive cues is an implicit process (Bartolomeo et al. 2007; López-Ramón et al. 2011; Risko and Stolz 2010). Furthermore, our results extend the pre-existing knowledge by showing that this implicit detection and use of the predictive cue is even possible for a very subtle manipulation like ours. In our experiment, different cue-target relationships prevailed in the left and right hemispace. This led to a fairly complex and, one might say, even obscure relationship between cue location and likely target location. Indeed, reports from our participants confirmed that most failed to determine the correct cue-target relationship (see Supplemental Analysis 1). Nevertheless, their performance demonstrated that the relationship was detected and used, albeit implicitly. The implicit nature of learning in this task might be beneficial for its application on neglect since it would not require any explicit insights of the patients into their attentional deficits and would hence circumvent the patients’ anosognosia (Grattan et al. 2018).

Lastly, as interpretations, as well as all future applications of the predictive cueing effect, rely heavily on participants truly learning about and responding to cue predictiveness, we wanted to ensure there were no other explanations for the observed effect. Hence, we assessed whether the detected RT benefits after left-sided cues predicting the right could correctly be attributed to our participants learning about the predictiveness of cues or whether the fact that targets, in total, appeared more often on the right drove the effect. Even though the bias for right-sided targets was rather subtle (in 57.5% of all trials, targets occurred on the right side but only in 42.5% on the left side), there is still a small chance that target occurrence statistics were detected by our participants and that they, hence, simply oriented their attention to the most likely (right) target side regardless of the cue (Peterson and Gibson 2011). Since we ultimately aim at implementing our task as an intervention for neglect patients, it is of special importance that we, firstly, know which mechanisms drive the potential therapeutic effect and, secondly, confirm that those mechanisms fit the difficulties neglect patients face in daily routines (i.e., the disengage deficit). The four-point configuration of our paradigm already made it less likely that participants oriented their attention to one specific target side instead of using cue predictiveness to locate the most likely target position. Our control analyses further corroborated that learning the cue predictiveness and, thus, learning attentional disengagement (and reorienting) were the driving factors behind our effect (see Supplemental Analysis 2). This is important as it suggests that the training indeed succeeds in modifying attentional disengagement and not only produces a general attentional bias to one side. While the latter would, of course, also be beneficial for patients with spatial neglect, the aim of this training is to target the disengage deficit in neglect patients in a side-specific manner, which no currently available treatment for neglect does.

Our study’s goal was to provide a proof of concept: We wanted to determine whether a side-specific modulation of attentional distribution by predictive cues was possible (and – to the tested extent – sustainable). We could confirm this with our data. However, it remains to be demonstrated that similar effects can also be obtained in neglect patients or patients with a disengage deficit in general and, furthermore, that such effects transfer to different contexts and, thus, also improve the clinical condition of the affected patients. Future studies will have to address those questions.

## Conclusion

In this study, we demonstrated that attentional disengagement and endogenous reorienting can be modulated side-specifically as well as implicitly in healthy, young participants and that this attentional modulation transfers to an uninformative cue setting. This study is but a first step in our long-term project to develop a treatment for neglect patients. A unilateral disengage deficit has been described as one of the key symptoms of unilateral neglect. Therefore, our paradigm, which aims specifically at modulating disengagement in one specific hemispace, holds promise as a novel treatment for patients with neglect. The hope is that patients will learn to disengage more effectively from ipsilesional stimuli and thus learn to shift their attention into their contralesional space. As neglect patients compared to healthy participants show similar albeit slowed predictive cueing effects after training with two-sided predictive cues, endogenous attentional orienting mechanisms do not seem to be severely impaired and could, thus, still be trained (Bartolomeo et al. 2001). Hence, there is reason to believe that – when given enough time – neglect patients could be trained in a similar way as our participants and that such a training could lead to a significant improvement of their disengage deficit, thereby possibly reducing their disability. Given that this training targets covert attention, it might complement existing neglect treatments.

## Electronic supplementary material

Below is the link to the electronic supplementary material.

Supplementary Material 1

## Data Availability

See Sect. “Data Analysis” in the methods section.

## Footnotes

1 A brief word about nomenclature: In the literature about the spatial orienting paradigm, cues that are followed by a target at the same location are usually called ‘valid’ cues, while those that are followed by a target at the opposite location are called ‘invalid’ cues (Chica et al. 2014; Posner 1980). Since in our paradigm some of those same cues have a predictive value (in the opposite direction), which one could also call ‘validity’ (i.e., a high-probability cue is ‘valid’, a low-probability cue is ‘invalid’), we will use the following naming convention: trials in which cue and target appear at the same location will be called ‘spatially congruent’, trials in which cue and target appear at opposite locations ‘spatially incongruent’. Hence, we will not talk about ‘validity effects’ but ‘spatial congruency effects’. Furthermore, whenever a cue contains a predictive value (meaning that following this cue the targets appear above chance level at a certain location. E.g., after cues on the left, 80% of the targets appeared on the right side during our intervention blocks), we will call it ‘predictive’. Thus, we hope to avoid the multiple possible meanings of ‘validity’ in the context of our experiment and instead use the terms ‘spatial congruency’ and ‘predictiveness’.
