# Supplementary material for: Side-specific implicit training of attentional disengagement and reorienting
Source: Exp Brain Res. 2025 May 28;243(7):156. doi: 10.1007/s00221-025-07049-0 (PMC12119381; doi:10.1007/s00221-025-07049-0)
Supplement: Supplementary file 1 — Supplementary Material 1 [file 221_2025_7049_MOESM1_ESM.pdf]

# Supplement

to “Side-specific Implicit Training of Attentional Disengagement and Reorienting”

Karin Ludwig, Raffaela M. M. Böswald, Johannes Schusterbauer, and Thomas Schenk

Clinical Neuropsychology, Department of Psychology, Ludwig-Maximilians-Universität München, Germany

## Supplemental Analysis 1: Implicitness of the Effect

We examined whether the one-sided predictiveness of cues was learned implicitly or whether an explicit perception and possibly an internal verbalization of the cue predictiveness was necessary to produce the effect. To this end, we determined how many participants reported awareness of a higher proportion of spatially incongruent cues in the debriefing questionnaire (see Supplemental Table 1). Eleven participants correctly guessed that cues had been more often spatially incongruent than congruent with the target; nineteen perceived cue predictiveness to be balanced; six participants assumed that cues had been more often spatially congruent than incongruent. We ran a one sample Chi-square test comparing these frequencies of statements in favour (11) and against (6) a preponderance of spatially incongruent cues (this means we did not include the 19 participants who perceived the cue predictiveness to be balanced during the whole experiment) and found that the frequencies did not differ significantly ($\chi^2(1) = 1.47$, $p = .23$).

This suggests that there was no clear evidence at the group level for awareness of the cue predictiveness, which was further supported by the following analysis: We examined our participants' numerical estimations of the proportion of spatially incongruent and congruent cues (second implicitness question in the debriefing questionnaire: “In how many trials (%) did the cue predict the opposite side for target appearance”) to check whether an overall explicit awareness about the predictiveness of the cues should be assumed in our sample. On average, participants underestimated

the proportion of spatially incongruent cues projected on all trials ($M$ = 51.97%, $SD$ = 11.86; real proportion: 57.59 %). A one-sided Wilcoxon signed-rank test showed that, on average, the numerical estimations of our participants did not significantly differ from 50% ($W$ = 91.50, $p$ = .49, $r_B$ = 0.004). A subsequent Bayesian one-sample Wilcoxon signed-rank test further revealed moderate support for the null hypothesis (BF01 = 3.93). Hence, the observed data were 3.93 times more likely under the assumption of the null hypothesis (50%) than under the alternative hypothesis (< or > 50%). As 50% reflects a balanced proportion of spatially congruent and incongruent cues, our results suggest that our participants, in total, were not aware of the real cue-target relationship.

Just in (the unlikely) case that the eleven participants who had correctly assumed that the proportion of spatially incongruent trials was above 50% were not just lucky guessers but did, in fact, have some awareness and drove the effect, we analyzed whether the effect was still present after their exclusion. The remaining sub-sample (of wrong guessers, $n$ = 25) still oriented their endogenous attention significantly faster after left-sided compared to right-sided spatially incongruent cues in the posttest ($DD$ = -16.55, $SD$ = 28.23) compared to the pretest ($DD$ = 2.99, $SD$ = 44.15; $W$ = 237, $p$ = .023, $r_B$ = 0.40; $t(24)$ = 2.01, $p$ = .028, $d$ = 0.40). Thus, we can assume that the detected predictive cueing effect is based on implicit learning or at least that the learned contingencies cannot be reported.

## Supplemental Analysis 2: Cue Predictiveness vs. Target Occurrence

Since the main aim of this study was to find out whether healthy participants can learn about one-sided cue predictiveness and use it to improve their attentional disengagement from one specific side, the interpretation of our results relies heavily on the fact that participants truly used the predictiveness of left-sided cues (of targets on the right) and nothing else to guide their attention. However, as the one-sidedness of predictive cues automatically leads to a higher proportion of right-sided targets (pretest: 40 out of 80, intervention: 156 out of 240, posttests: 80 out of 160, in total: 276 out of 480 targets (57.5%)), we needed to consider the possibility that participants automatically

oriented their attention to the right side due to target side regularities but did not pay attention to the cue predictiveness. As a first step to make this less likely, we used the experimental paradigm with four instead of two positions to give the cue additional information (see section 1.5).

To further examine whether the effect was truly produced by the learned cue predictiveness and not by learned target occurrence statistics, we added another analysis focusing only on trials with right-sided cues (which were uninformative throughout the whole experiment). If participants automatically oriented their attention to the right side regardless of the cue, their RTs to right-sided targets after right-sided cues would also speed up in the intervention compared to the pre-test, even though right-sided cues never changed in their predictiveness. To test this prediction, we performed a 2x2 ANOVA with the factors 'Time' (Pre, Int) and 'Target Side' (left, right) based only on trials with right-sided cues. The ANOVA revealed significant results for both main factors (time: $F(1,35) = 10.07$, $p = .003$, $\eta_p^2 = .22$; side: $F(1,35) = 31.20$, $p < .001$, $\eta_p^2 = .47$). The main effect of time can be explained by practice effects and the main effect of side by the fact that trials with targets on the left were spatially incongruent and those with targets on the right spatially congruent. Crucially, however, the analysis failed to yield a significant result for the interaction ($F(1,35) = 0.25$, $p = .622$, $\eta_p^2 = .007$). This can also be seen in the almost parallel lines in Supplemental Figure 1.

**Supplemental Figure 1**

*Mean Reaction Times in the Pretest and Intervention after right-sided cues only*

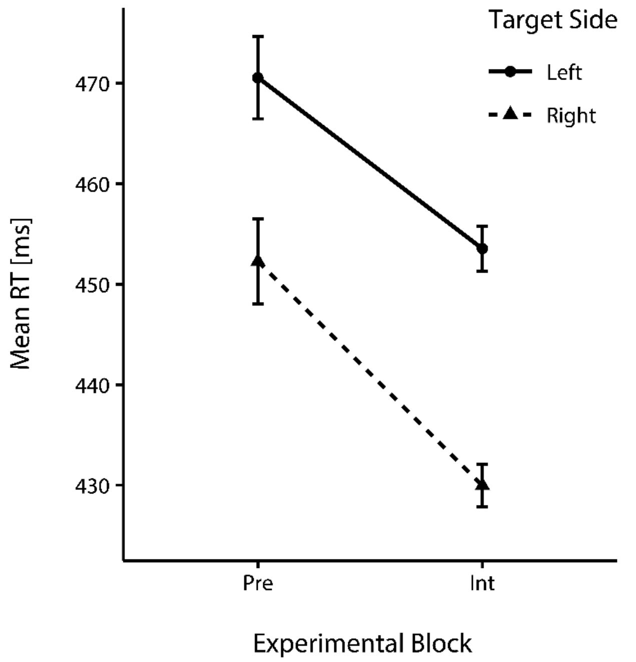


*Note.* Mean RTs in the pretest (Pre) and the intervention block (Int) are split by target side. Since only trials with cues on the right were analyzed, trials with targets on the left were by definition spatially incongruent, and target-right trials spatially congruent.

This points to the fact that the factor 'Target Side' is independent of the factor 'Time', which is in favor of our hypothesis that participants did not react according to target side probabilities in the intervention block but to cue predictiveness.

To examine to what extent the data support the null hypothesis, we used the program JASP (JASP Team, 2020; Wagenmakers et al., 2018) to carry out a Bayesian two-way repeated-measures ANOVA with a default prior for the mean RTs (Rouder et al., 2012) and the factors 'Time' (Pre, Int) and 'Target Side' (left, right). As in the frequentist ANOVA, we compared mean RTs only for trials with right-sided cues. The Bayesian ANOVA revealed a highly influential factor 'Time' (BF10 = 82.880) which was, as depicted in Figure 5, driven by overall faster RTs in the intervention block. Adding the second factor, 'Target Side' to the model (BF10 = 3852.14, time + target side: BF10 = 1,531,000) increased the support by a factor of 16,300 (1,531,000/ 82.880). However, adding the interaction term

to the model (BF10 = 1,286,000) reduced the support by a factor of 1.05 (1,531,000/1,286,000). Therefore – according to Jeffreys' classification (1998) modified by Lee and Wagenmakers (2013) – the data provide, at best, anecdotal evidence against including the interaction term. To fully support the null hypothesis, the Bayes analysis would have needed to reject the inclusion of the interaction term; with a factor of 1.05 we lack the evidence for a decision for H1 or H0.

However, a more sensitive Bayesian paired sample t-test comparing single difference values ($D = RT_{incongrunet} - RT_{congruent}$) for trials with only right-sided cues in the pretest ($D_{Pre}$= 20.82, *SD* = 31.95) and the intervention ($D_{Int}$= 23.40, *SD* = 24.35) revealed moderate support for the null hypothesis (BF01 = 4.975, error probability <0.001). Hence, the observed data were 4.975 times more likely under the assumption of the null hypothesis than under the alternative hypothesis. Therefore, the Bayesian analysis indicated that the predictive cueing effects did not differ between the pretest and the intervention; see also the Bayes Factor robustness check in Supplemental Figure 2. From this Bayesian analysis, we can conclude that participants did not react towards target side regularities but used the cue predictiveness to orient their attention accordingly.

**Supplemental Figure 2**

*Bayes factor robustness check*

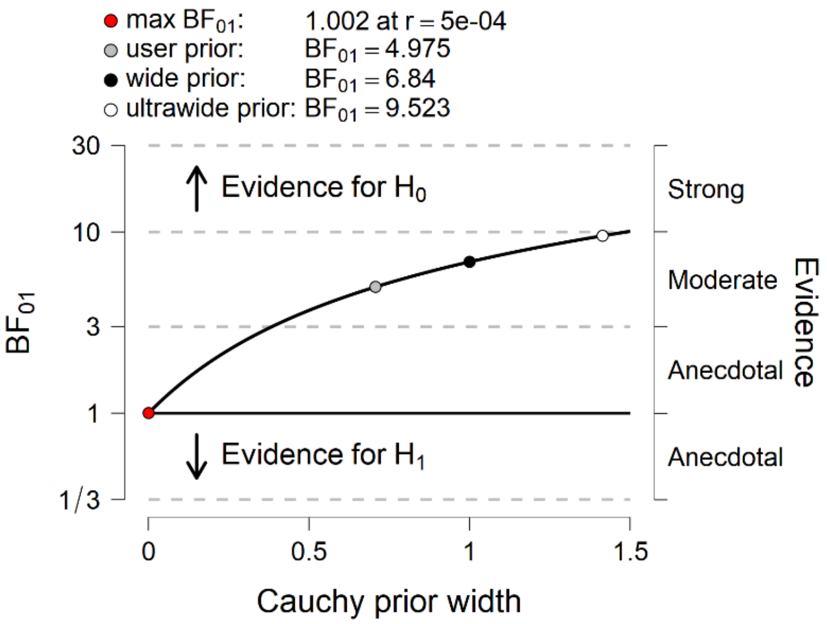


*Note.* Bayes factor robustness check for the Bayesian paired sample t-test comparing single difference values ($D = RT_{invalid} – RT_{valid}$) for trials with only right-sided cues. For a wide range of priors, we found moderate evidence in favor of the $H_0$, indicating that the findings in favour of the null hypothesis are relatively robust.

# Supplemental Material

**Supplemental Table 1**

*Template of the Debriefing-Questionnaire in German (Original) and English (Translation).*

| German Version (Original) | English Translation |
|---|---|
| 1. Was vermutest Du, wurde bei diesem Experiment untersucht? | 1. What do you think was investigated in this study? |
| 2. Ist Dir beim Experiment irgendetwas Bestimmtes aufgefallen? | 2. Did you notice anything special during the experiment? |
| 3. Ist Dir bzgl. der Übereinstimmung der Seite (rechts/links) des Hinweisreizes (Aufblinken des Kästchens) und der Seite des Zielreizes (X bzw. O) etwas aufgefallen? | 3. Did you notice anything concerning the accordance of the side (right/left) of the cue (highlighting of one of the boxes) and the side of the target (X or O)? |
| 4. Hat der Hinweisreiz häufiger die gleiche oder die entgegengesetzte Seite für einen Zielreiz vorhergesagt oder war es ausgeglichen? | 4. Did the cue more often predict the same or the opposite side for target appearance or was it balanced? |
| 5. Wie oft schätzt Du (%) erschien der Zielreiz auf der entgegengesetzten Seite des Hinweisreizes? | 5. Please estimate: In how many trials (%) did the target appear opposite to the cue? |
| 6. Wenn Du ein Verhältnis (r/l) schätzen könntest, in dem der Zielreiz auf der rechten bzw. linken Seite erschienen ist, welches wäre es? | 6. If you had to estimate the ratio of the target appearing on the right or the left side, respectively, what would you say? |

*Note.* Participants answered the questionnaire (German Version) orally. Participants' statements concerning questions four and five were used to examine the implicitness of the manipulated counter-predictiveness. The English version was not used in the experiment.

# Supplemental References

JASP Team. (2020). *JASP (Version 0.12.2) [Computer software]*. In https://jasp-stats.org/

Jeffreys, H. (1998). *The theory of probability*. OUP Oxford.

Lee, M. D., & Wagenmakers, E.-J. (2013). *Bayesian cognitive modeling: A practical course*. Cambridge University Press. https://doi.org/10.1017/CBO9781139087759

Rouder, J. N., Morey, R. D., Speckman, P. L., & Province, J. M. (2012). Default Bayes factors for ANOVA designs. *Journal of Mathematical Psychology*, *56*(5), 356-374. https://doi.org/https://doi.org/10.1016/j.jmp.2012.08.001

Wagenmakers, E.-J., Love, J., Marsman, M., Jamil, T., Ly, A., Verhagen, J.,…Morey, R. D. (2018). Bayesian inference for psychology. Part II: Example applications with JASP. *Psychonomic Bulletin & Review*, *25*(1), 58-76. https://doi.org/10.3758/s13423-017-1323-7
